# The Role of Porcelain Veneers in the Aesthetic Restoration of Discolored Endodontically Treated Teeth

**DOI:** 10.3390/clinpract14050164

**Published:** 2024-10-09

**Authors:** Panagiotis Galiatsatos, Aristidis Galiatsatos

**Affiliations:** Division of Dental Technology, Department of Biomedical Sciences, University of West Attica, 12243 Athens, Greece; pgaliatsatos@uniwa.gr

**Keywords:** porcelain veneer, aesthetic, discolored teeth, restoration, endodontically treated teeth

## Abstract

Background: The discoloration of endodontically treated anterior teeth poses a significant aesthetic concern for many individuals, impacting their confidence and self-image. Porcelain veneers have emerged as a popular solution for the aesthetic restoration of such teeth. This paper explores the role of porcelain veneers in addressing tooth discoloration, examining their efficacy, durability, and aesthetic outcomes via a clinical case. Case description: In this clinical case, an aesthetic restoration of a discolored central incisor was performed using a ceramic veneer. Due to the high degree of discoloration, an internal bleaching of the tooth was carried out prior to the final restoration. Various factors influencing the selection of porcelain veneers as a treatment modality, including shade matching, preparation techniques, and adhesive bonding, are discussed. Conclusions: The advancements in materials and techniques have enhanced the versatility and aesthetic appeal of porcelain veneers, making them a valuable option for achieving natural-looking and durable aesthetic restorations in individuals with discolored endodontically treated anterior teeth.

## 1. Introduction

Many different materials and treatment options are available in restorative aesthetic dentistry for the anterior aesthetic zone. One of the most common therapeutic approaches is the use of porcelain veneers. The high aesthetic performance, excellent biocompatibility of porcelain materials, advancements in bonding techniques with dental tissues, and the longevity of restorations have made porcelain veneers a reliable treatment solution for the anterior aesthetic zone, despite being a sensitive clinical and laboratory process [1,2,3,4,5,6,7]. The use of veneers is primarily indicated for restoring anterior teeth with abrasions, fractures, tooth decay, enamel defects, hypoplasia, or replacing old and extensive resin composite fillings that have been retreated or do not have satisfactory aesthetic results. Porcelain veneers can also be used to change the shape of teeth or close spaces, often in combination with orthodontic treatment. Additionally, they provide aesthetic solutions for cases of discolored teeth due to tetracycline use, fluorosis, or endodontic treatment. The use of porcelain veneers for the aesthetic restoration of discolored endodontically treated teeth in the anterior aesthetic zone, although not a very common technique, is gaining ground in aesthetic dentistry due to its conservative nature compared to placing a full crown [8,9,10,11]. In such clinical cases, an external bleaching of the discolored tooth usually precedes the final restoration in order to achieve a better aesthetic outcome. This paper presents the use of porcelain veneers for the aesthetic restoration of endodontically treated teeth that have undergone discoloration, examining their efficacy, durability, and aesthetic outcomes, via a clinical case. The main characteristic of this clinical case is that, prior to the final restoration, an internal bleaching of the affected tooth was performed due to the severe existing discoloration.

## 2. Clinical Case

**Figure 1:**
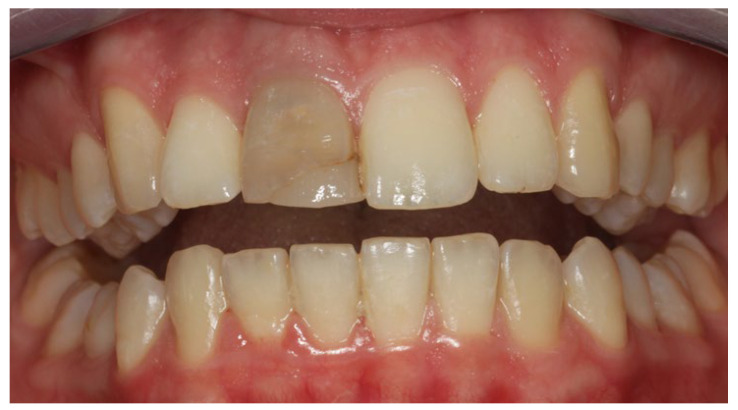
Intraoral photograph at the first visit. The discoloration on tooth No #11 is evident, causing an aesthetic issue.

A 43-year-old woman presented at the private dental clinic due to the discoloration of tooth #11. This tooth also had an old composite resin filling that needed replacement (Figure 1).

**Figure 2:**
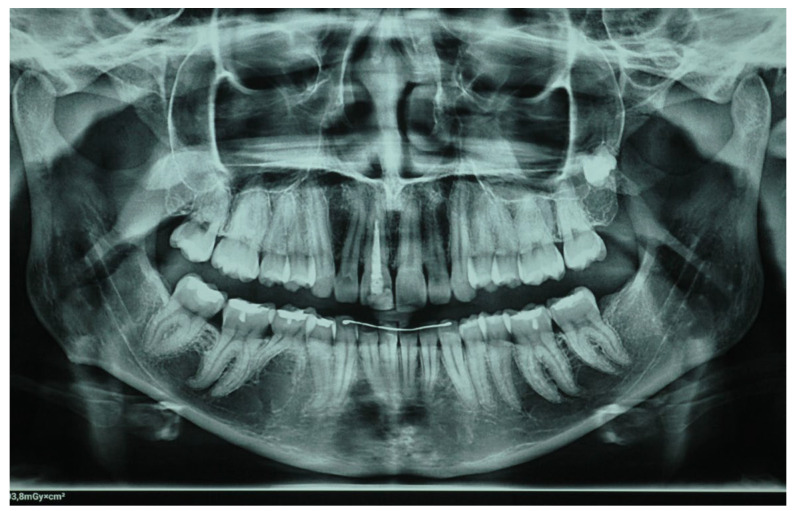
The panoramic X-ray of the patient.

The patient reported that this tooth had undergone an endodontic treatment five years ago. The patient’s general medical history was clear, with no underlying diseases. The overall clinical condition of the patient’s teeth was good, and the condition of the periodontal tissues was satisfactory, with no obvious signs of periodontal disease or inflammation. Additionally, the clinical examination revealed that the patient had no parafunctional habits. A panoramic X-ray examination was performed to assess the quality of the endodontic treatment of the specific tooth, which was found to be satisfactory (Figure 2).

The patient was presented with all possible treatment options, including internal whitening as well as a composite resin veneer, a porcelain veneer, or a full crown, and it was ultimately decided to restore the specific discolored tooth with a porcelain veneer, following internal bleaching to reduce the degree of discoloration.

**Figure 3:**
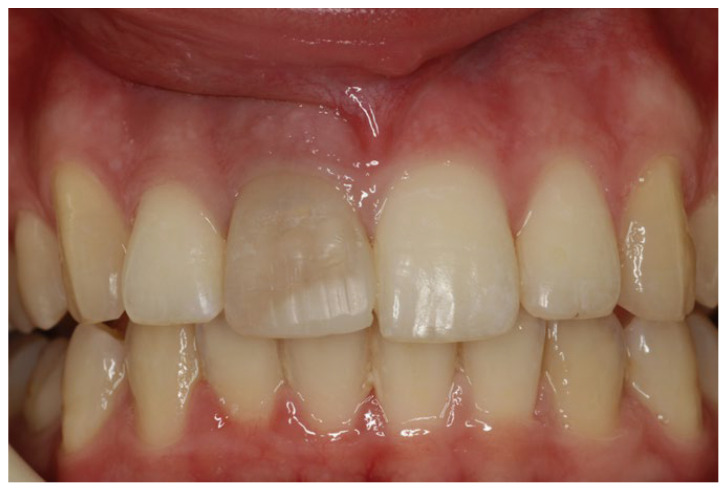
The final result after the internal bleaching and the replacement of the old filling on tooth No #11.

The tooth was opened on the palatal surface, and the pulp chamber was cleaned of the remnants of the endodontic treatment material. After sealing the root canal orifice with glass ionomer cement (Table 1, GC Fuji II LC, GC Corporation, Tokyo, Japan), a ready-to-use bleaching gel (Table 1, BMS white 38%, active ingredient: 38% hydrogen peroxide, BMS Dental S.r.I., Capannoli, Italy) was applied directly into the pulp chamber using a special syringe, and the cavity was hermetically closed with a temporary filling material (Table 1, Provis, Favodent Berlin GmbH, Berlin, Germany). The entire procedure was performed with a rubber dam placed on the affected tooth due to the bleaching agent’s potential risk to the soft tissues. Four days later, the patient was recalled to the clinic to assess the progress of the bleaching. During the recall appointment, the temporary filling was removed, the cavity was cleaned of the bleaching agent, and then the bleaching agent was reapplied, followed by a temporary filling for an additional 3 days. The final result of the internal bleaching is depicted in Figure 3.

After the internal bleaching procedure, the old composite resin filling was replaced with a new one (Figure 3), followed by the preparation of the tooth for a porcelain veneer. Before the preparation, a diagnostic wax-up was performed to serve as a reference point. The preparation was limited to the buccal surface, and the amount of tooth substance removal was kept to a minimum. Specifically, no more than 0.5–1 mm of the tooth structure was removed to preserve the enamel across almost the entire preparation, ensuring both mechanical retention and bonding capability with the porcelain veneer. Preparation was performed using the guided depth cutting technique with L.V.S. diamond burs (Table 1, Laminate Veneer System, Komet Co, Fort Mill, SC, USA). The cervical margin of the preparation was defined at the gingival level in a chamfer shape. The preparation extended towards the adjacent surfaces but stopped short of the contact points with the neighboring teeth to preserve and keep them intact. Additionally, there was a reduction in the incisal edge by 1.5 mm through a reverse beveling of the enamel. This created a larger enamel surface, necessary for better bonding and resistance in an area subject to significant stress. Finally, all undercuts and sharp edges were smoothed out during the final stage of preparation.

**Figure 4:**
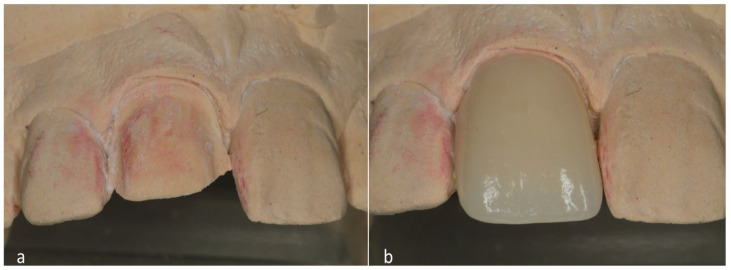
(**a**): The master model with the prepared tooth, (**b**): the final restoration on the master model.

After the preparation was completed, the teeth were scanned with a chairside CAD/CAM system (Emerald S Intraoral Scanner, Planmeca, Helsinki, Finland) and built-in software (PlanCAD Easy 6.2, Planmeca, Helsinki, Finland). A third-generation zirconia (5Y-YZP) veneer to be used for the restoration (Table 1, Katana UTML, Kuraray Noritake, Tokyo, Japan) was milled with a dental laboratory milling machine (PrograMill PM7, Ivoclar Vivadent, Schaan, Liechtenstein). The veneer was glazed (Cerabien ZR FC, Kuraray Noritake, Tokyo, Japan), sintered in a furnace (Programat S2, Ivoclar Vivadent, Schaan, Liechtenstein), and polished (Table 1, Zir-Cut, Zirconia Polisher Kerr Corporation, Brea, CA, California, USA), following the manufacturer’s recommendations (Figure 4a,b).

**Figure 5:**
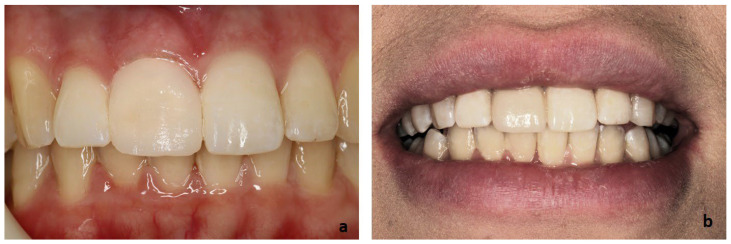
The final outcome of tooth No. #11 after the completion of all clinical procedures: (**a**): frontal view, (**b**): patient’s smile.

After the fabrication of the zirconia veneer, it was clinically evaluated to assess its marginal fit and aesthetic outcome. Despite the satisfactory aesthetic and color result, there was a small asymmetry between the left and right central incisors (Figure 5a). It was suggested to the patient to further improve the aesthetic appearance with the construction of a veneer on the adjacent central incisor as well, but the patient refused any further intervention because she wanted to be conservative. Thus, the bonding of the veneer to the patient’s tooth followed. The zirconia veneer was prepared for bonding with 50 mm Al_2_O_3_ airborne-particle abrasion at 0.1 MPa air pressure for 10 s. A porcelain primer containing silane and an MDP monomer (Table 1, Monobond Plus, Ivoclar Vivadent AG, Schaan, Liechtenstein) was applied to the veneer and then dried with oil-free air. The enamel tooth surfaces were treated with 37% phosphoric acid (Table 1, Total Etch Gel, Ivoclar Vivadent AG, Schaan, Liechtenstein) for 15 s, rinsed with water, and then airdried. A bonding agent (Table 1, Adhese Universal, Ivoclar Vivadent AG, Schaan, Liechtenstein) was applied and light polymerized for 10 s. Light-polymerized resin cement (Table 1, Variolink Esthetic LC, Ivoclar Vivadent AG, Schaan, Liechtenstein) was applied with the applicator tip to the intaglio of the veneer and evenly spread with a microbrush. The restoration was placed on the tooth and seated under firm finger pressure to achieve the desired cement thickness. An oxygen-inhibiting gel (Table 1, Liquid Strip, Ivoclar, Vivadent AG Schaan, Liechtenstein,) was applied after the excess cement was removed. The natural and aesthetic appearance of the prostheses was very satisfactory for the patient (Figure 5a,b).

The table below summarizes all the materials used in this clinical case.

**Table 1:** Materials used in the case report.

MATERIAL	TRADE NAME	COMPANY
Glass ionomer cement	GC Fuji II LC	GC Corporation, Tokyo, Japan
Bleaching gel	BMS white 38%	BMS Dental S.r.I., Capannoli, Italy
Temporary filling material	Provis	Favodent Berlin GmbH, Berlin, Germany
Burs and Diamonds	L.V.S. diamond burs Laminate Veneer System	Komet Co, Fort Mill, South Carolina, USA
Third-generation zirconia	Katana UTML	Kuraray Noritake, Tokyo, Japan
Zirconia Polisher	Zir-Cut	Kerr Corporation, Brea, CA, California, USA
Porcelain primer	Monobond Plus	Ivoclar Vivadent AG, Schaan, Liechtenstein
37% phosphoric acid	Total Etch Gel	Ivoclar Vivadent AG, Schaan, Liechtenstein
Bonding agent	Adhese Universal	Ivoclar Vivadent AG, Schaan, Liechtenstein
Light-polymerized resin cement	Variolink Esthetic LC	Ivoclar Vivadent AG, Schaan, Liechtenstein
Oxygen-inhibiting gel	Liquid Strip	Ivoclar Vivadent AG, Schaan, Liechtenstein

## 3. Discussion

Treating aesthetic and functional issues of the aesthetic zone with porcelain veneers is one of the most prevalent therapeutic approaches. Porcelain veneers offer a predictable solution for conservative restorations of the facial surface of anterior teeth, serving as an alternative to full crowns and direct composite resin restorations. Their main advantages include excellent biocompatibility, high aesthetic performance, and the conservative nature of tooth preparation.

One indication for porcelain veneers is the aesthetic restoration of discolored teeth. The presence of discolorations in the anterior aesthetic region of the dental arches constitutes one of the most significant aesthetic problems and concerns an increasing per-centage of patients nowadays. They have a negative impact on self-esteem, external appearance, interpersonal relationships, and the projection of one’s image to the social environment [12,13]. Endodontically treated teeth often exhibit changes in color, particularly in the challenging area of the clinical cervical region, leading dentists to opt for the restoration of these teeth with full-coverage crowns [14,15]. Porcelain veneers provide a reliable and conservative alternative in these cases, as there is no need for the preparation of the palatal or lingual surfaces of the teeth, while the preparation of the facial surface is limited to 0.5–1 mm.

The main characteristic of this clinical case is that, prior to the final restoration, internal bleaching of the affected tooth was performed due to the severe existing discoloration. For better aesthetic results, in cases of intrinsic discoloration, an internal tooth bleaching procedure may precede, as was performed in this specific case. Applying bleaching techniques to discolored endodontically treated teeth achieves their decolorization thus facilitating the dentist in their aesthetic restoration. Bleaching endodontically treated teeth is a minimally invasive solution for improving their color [14,15,16]. The materials causing discoloration typically comprise organic residues with extensive carbon chain monomers or dimers, often containing complexes of atoms from other chemical elements and phenylic and carbonylic rings, generally referred to as chromophore groups. Bleaching agents act as oxidative agents on these chromophore groups, modifying the polarity and shape of the discolored molecules [17]. Clinical examination should be accompanied by radiographic evaluation of endodontic treatment. Root canal obturation should be adequate in height and width to prevent bacterial microleakage and the spread of bleaching agents to periapical tissues [18,19]. Endodontically treated teeth should be asymptomatic, with the absence of clinical and radiographic findings indicative of periapical lesion development [19]. Τhe application of hydrogen peroxide (H_2_O_2_) for bleaching endodontically treated teeth was first reported by Harlan [20]. The mechanism of action of hydrogen peroxide is quite complex. Its target molecules are the chromophoric organic compounds responsible for discoloration. Its strong oxidizing action leads to the formation of active oxygen radicals and peroxide anion compounds. The oxidation products act on the chromogenic compounds, breaking them down into smaller, colorless, and more soluble molecules [19,20,21,22].

There are many clinical studies that confirm the positive outcomes of internal bleaching in discolored endodontically treated teeth, as evidenced in this specific clinical case. Zimmerli B et al. stated that the bleaching of nonvital, discolored teeth is a low-risk, standard procedure that enhances aesthetics. The internal bleaching technique is relatively dependable and fairly straightforward for both dentists and patients [23]. Pedrollo Lise et al. stated that for patients with discolored endodontically treated teeth, intracoronal bleaching serves as a conservative aesthetic treatment that yields good aesthetic outcomes. Additionally, this technique is a simple and cost-effective approach that typically produces noticeable and satisfactory results [24]. Amato A et al. stated that 10% carbamide peroxide proved to be an effective dental whitening agent in the long term for endodontically-treated [25]. In another study, Glockner K et al. stated that the internal bleaching method is an acceptable technique for lightening discolored anterior teeth. This follow-up study revealed a success rate of 79% for all indications after 5 years. The results clearly demonstrate that internal bleaching achieves long-term success in treating discolored nonvital anterior teeth over several years, without causing any harmful effects on dental hard tissue [26].

Various types of porcelain materials are recommended for making veneers, including lithium disilicate, feldspathic porcelain, feldspathic porcelain reinforced with leucite, and lithium silicate reinforced with zirconia [27,28,29,30]. These porcelains are prized for their high translucency, owing to their abundant glass matrix content, which ensures pleasing aesthetics. Moreover, they exhibit strong adhesion to adhesive agents following conditioning with hydrofluoric acid (4–10%) and subsequent silanization [31]. Consequently, these porcelains are preferred for veneer production [27,28]. Nonetheless, they come with certain limitations, particularly their inability to effectively conceal significant dental discolorations and their increased fragility when reduced in thickness [29].

Alternatively, porcelains with a high crystalline content, such as yttria-stabilized tetragonal zirconia polycrystals (5Y-YZP) were initially primarily utilized for framework fabrication, given their notable fracture resistance and capacity to conceal substrate discolorations [31]. However, computer-aided design and computer-aided manufacturing (CAD-CAM) technology and the improved translucency of recently developed high strength monolithic zirconia have made them clinically acceptable for bonded restorations [27,28]. The higher translucency is achieved by altering the grain size and sintering temperature and by adding more yttria to reduce the residual pores and reduce the impurities [31,32]. Also, in recent years, several manufacturers have enhanced the composition of Zr to create a polychromatic multilayer, aiming to mimic the shade gradient observed in natural teeth. This design involves the incisal portion of the veneer being the most translucent, with chroma and opacity increasing gradually towards the gingival portion [33,34]. Initially, the pre-shade layers of the same Zr composition were created to form a polychromatic multilayer, presenting as uniform Zr. It has been indicated that the only difference among the various layers of this uniform multilayer zirconia material is the pigment composition, leading to distinct shades while maintaining similar translucency [33,34]. Following that, ultra-translucent zirconia (Zr) materials were introduced, featuring distinct microstructures among the layers. These included tetragonal zirconia polycrystals (TZP) and partially stabilized zirconia (PSZ) layers with diverse compositions and properties, resulting in the development of a polychromatic multilayer hybrid composition of zirconia [35,36]. The varying Yttrium content and chemical composition of the layers result in diverse formulations, leading to differences in the physical properties within the material. Consequently, several manufacturers suggest that advancements in the different grades of ultra-translucent zirconia have made monolithic translucent zirconia a feasible choice for restoring anterior teeth with indirect veneer restorations [36,37].

In contrast to lithium disilicate veneers, achieving strong bonding to zirconia has presented greater challenges. Polycrystalline zirconia is chemically inert and not susceptible to attack by hydrofluoric acid, resulting in less effective adhesion compared with silica-based porcelains [38]. So, studies have indicated that a combination of micromechanical and chemical pretreatment is essential for establishing durable resin bonds with zirconia restorations over the long term. Airborne-particle abrasion using Al_2_O_3_ is effective in creating mechanical bonds, while an adhesive resin cement or a porcelain primer containing monomers like 10-methacryloyloxydecyl dihydrogen phosphate (MDP) is advised for achieving chemical bonding to zirconia [38,39,40,41,42]. According to research by Blatz et al., the APC Zr Bonding Concept is a useful technique for creating strong, durable resin bindings to Zr [42]. The following three primary processes of zirconia cementation are referred to as the APC Zirconia Bonding Concept [42]:

Step A: The process entails air-abrading the entire surface of the zirconia intended for bonding. This is achieved using either plain alumina particles or alumina particles coated with silica.

Step B: MDP or phosphate-monomer-based primer is applied to the air-abraded zirconia surfaces.

Step C: Utilizes a dual-cure adhesive composite to guarantee thorough polymerization of the composite beneath the zirconia restoration.

A case series with a follow-up period of up to five years has shown a 100% survival rate for ultra-translucent zirconia veneers bonded with adhesive resin cement after airborne particle abrasion and silica coating. No noticeable failures such as debonding, veneer fracture, or secondary caries were observed [43,44,45]. Additionally, when bonded with a resin-based adhesive system, the fracture resistance of monolithic all-porcelain restorations is increased compared to conventional cementation methods [43,46].

## 4. Conclusions

Based on all the aforementioned information, we can draw the following conclusions:

- Porcelain veneers provide a reliable and conservative procedure for the aesthetic restoration of endodontically treated teeth that have undergone discoloration.
- For better aesthetic results, in cases of intrinsic discoloration, an internal tooth bleaching procedure may precede.
- Considering bonding and cementation, adhesively cemented high translucency monolithic zirconia seems to be a suitable option for veneers.

## Data Availability

The data presented in this study are available on request from the corresponding author due to ethical reasons.
